# Evaluating Potential Behavioral Mediators for Increasing Similarity in Friends’ Body Size among College Students

**DOI:** 10.3390/nu11091996

**Published:** 2019-08-23

**Authors:** Irene van Woerden, Daniel Hruschka, David R. Schaefer, Kimberly L. Fine, Meg Bruening

**Affiliations:** 1 College of Nursing, Idaho State University, Pocatello, ID 83209, USA; 2 School of Human Evolution and Social Change, Arizona State University, Tempe, AZ 85281, USA; 3 Department of Sociology, University of California—Irvine, Irvine, CA 92697, USA; 4 Department of Applied Health Science, School of Public Health-Bloomington, Indiana University, Bloomington, IN 47405, USA; 5 College of Health Solutions, Arizona State University, Phoenix, AZ 85004, USA

**Keywords:** college, social network, friends, diet, physical activity, sleep, weight-related behaviors, BMI, longitudinal

## Abstract

College students and their friends become more similar in weight status over time. However, it is unclear which mediators explain this relationship. Using validated survey measures of diet, physical activity, alcohol intake, sleep behaviors, mental health, and food security status, we take a comprehensive look at possible factors associated with excess weight gain that may explain friends’ convergence on body mass index (BMI), waist circumference, waist to hip ratio, and waist to height ratio over time. We use linear mixed models applied to a longitudinal dataset of first-year college students to examine whether these variables satisfy two criteria for potential candidate mediators of friends’ influence on anthropometrics—cross-sectional similarity among friends (n = 509) and longitudinal associations with increasing anthropometrics (n = 428). While friends were similar on some survey measures (such as dining hall use, home cooked meal consumption, fruit intake, alcohol intake, hours of sleep, and stress). Only dining hall use and stress emerged as potential explanations for why friends’ BMI and anthropometric change may be similar. Given that only a few variables satisfied the two criteria as potential mediators, future research may need to consider alternative measurement approaches, including real-time assessments, objective measurements, and alternative factors causing the convergence of friends’ and college students’ body size over time.

## 1. Introduction

Weight gain during college remains a persistent health issue [1,2,3]. Despite the claim of the “freshman 15” being debunked, weight gain throughout college remains a concern, with many students gaining 3–5 pounds during the freshman year [4,5]. On average, weight gain during college predicts weight gain later in life [6]. While there are biological and environmental influences on obesity [7,8,9,10], behaviors have been identified as critical components to obesity prevention and treatment [11,12]. For example, college students appear to have a lower risk of weight gain and obesity if they consume a diet that is plant-rich, low in sugar-sweetened beverages, and low in fast foods [13]. College students who participate in recommended amounts of physical activity (PA) also have lower reported body mass indexes (BMIs) [14,15,16]. In addition to diet and physical activity, there is increasing interest in the role that alcohol [17], sleep [18,19] and stress [20] have on weight gain and obesity risk among college students and other populations. Vulnerable students, such as those who are food insecure, may also be at greater risk for weight gain during college [21,22,23]. Understanding the mechanisms by which these factors influence energy balance and subsequent risk for excess weight gain during college years is critical for obesity prevention efforts.

It has been suggested that obesity can be conceptualized as a communicable disease [24,25,26]; an emerging body of evidence suggests that friends have the potential to influence weight gain across the life course [24,27,28,29,30]. In one recent study, we reported that among diverse college students, friends influenced one another’s increases in BMI over time. Specifically, students whose friends had higher BMIs compared to themselves had 2.85 higher odds of increasing their own BMI over time, relative to students whose friends had a lower or same BMI. These findings were consistent even after controlling for the selection effect of students who preferred to befriend those who did not have an extreme BMI (either underweight or obese) [29]. Despite increasing evidence that friends influence each other’s weight gain, the behavioral mechanisms underlying this influence are not yet understood [31].

A paucity of studies has examined how contagion of weight-related behaviors may mediate friends’ influence on BMI. Several cross-sectional studies have reported similarities in weight-related behaviors such as eating and PA among friends. For example, de la Haye et al. reported similarities among adolescent friends for PA, screen-time behaviors, and high calorie foods [32]. A longitudinal study conducted by Ali et al. found a positive association between adolescents and their friends’ PA and fast food consumption behaviors [33]. While these rare studies have identified a number of potential factors underlying friends’ influence on BMI, even fewer studies have linked shared behavior among friends to BMI and weight outcomes over time. Madan et al. examined weight-related behaviors (five questions on fruit and vegetable intake, perceived healthfulness of diet, and aerobic and sport frequency) among social networks and the effects on weight gain in a college population [34]. While the authors reported convergence of some behaviors (e.g., unhealthy eating habits) over time by peers (as measured by Bluetooth) among 70 students in a residence hall, the authors did not observe similarities in eating and PA behaviors nor weight change as a result of convergence with friends. However, given that weight was self-reported and measures of diet and PA were not validated, measurement imprecision may have been a source of these null findings.

Given the consistent finding that friends influence weight status [29,35], and continuing uncertainty about the mechanisms underlying this influence, we use a 4-wave longitudinal study design to assess a comprehensive set of candidate behavioral mechanisms potentially underlying friends’ influence on weight gain. If friends influenced each other’s weight through behavioral mechanisms, friends would be anticipated to be similar on the candidate behavioral mechanisms, and the behavioral mechanisms would be anticipated to be associated with weight/weight gain. In this study, we first examine if friends’ weight-related behaviors are similar at several points in time, and then examine if the weight-related behaviors are associated with students’ future weight and weight gain. Studying the potential convergence in weight-related behaviors and weight change is ideal in college student populations, as their behaviors and social networks are changing rapidly, and potentially concurrently.

## 2. Materials and Methods

### 2.1. Study Design and Population

**Figure 1:**
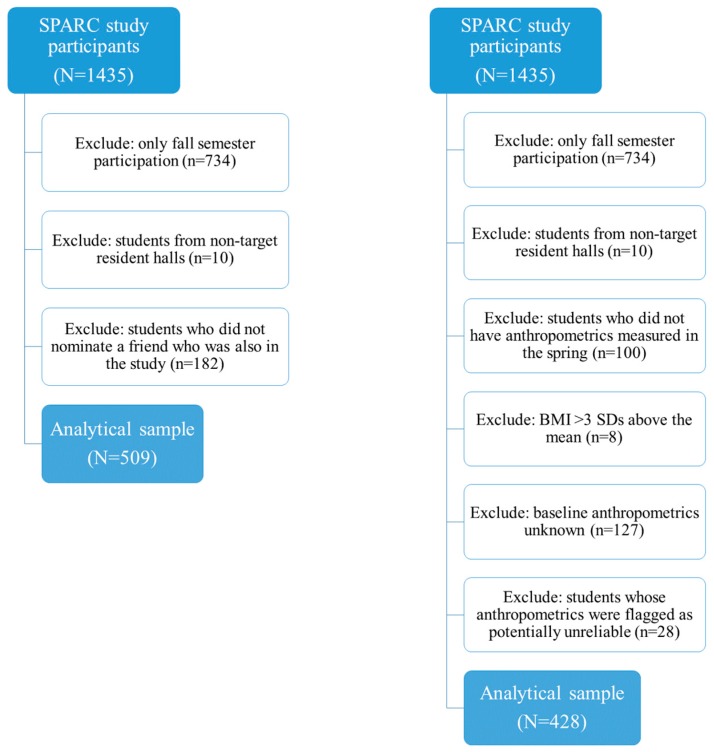
Flowchart of the study population and sample size for the analysis of friend similarities and behaviors (**left**), and behavioral associations with anthropometrics (**right**).

The Social impact of Physical Activity and nutRition in College (SPARC) study was a longitudinal study which aimed to determine how students’ friendship networks were associated with nutrition, physical activity, and weight gain [36]. A total of 1435 students from a large, public, southwestern university participated in the SPARC study during the 2015–2016 academic year. Recruitment was targeted at the first-year college students, and their resident assistants, from six residence halls; anyone from these residence halls and the targeted first-year floors were invited to participate. For the study examining friendship similarities, inclusion criteria included being a resident at one of the targeted resident halls and nominating a friend who was also a participant in the study. For the analyses examining anthropometrics, inclusion criteria included being a resident at one of the targeted residence halls, and having complete anthropometric measurements. For both analyses, students who only completed surveys in Fall were excluded from the analyses. Students completed web-based surveys at the start and end of the Fall 2015, and Spring 2016, semesters. Trained research staff measured students’ weight, height, waist circumference, and hip circumference at the same approximate time the surveys were completed, typically within 24 h. Similarities among friends would be expected to be greater in the Spring semester (nearing the end of the academic year) than in Fall if behavior convergence among friends occurred. As such, only the similarities among friends for the survey responses in the Spring 2016 semester were examined. A total of 691 students from the target residence halls completed a survey at the start and/or end of the Spring 2016 semester. To maximize power, two different analytical samples were used in these analyses. For the cross-sectional analyses examining friend similarity in behaviors, students who did not nominate a friend (n = 182) were excluded, resulting in a sample of 509 students. For the longitudinal analyses examining the behavioral associations with anthropometrics, students whose anthropometrics were not measured at the time of the survey (n = 100), students with a BMI greater than 3 standard deviations from the mean (n = 8), students with unknown baseline anthropometrics (n = 127), and students whose anthropometrics were flagged as potentially unreliable (n = 28), were excluded, resulting in a sample of 428 students. See Figure 1 for a flowchart showing how both sample sizes were obtained. Given that some students had incomplete surveys, the analytical sample used in each model is slightly less than base analytical samples of 509 and 428. The Arizona State University IRB approved all study protocols (approval number: 1309009596).

#### 2.1.1. Measurements

Students’ diet, physical activity, alcohol intake, sleep behaviors, mental health, and food security status were assessed in each survey (start and end of Fall 2015 and Spring 2016 semesters) using validated measures. Students also completed a friendship nomination questionnaire [36], as reported elsewhere [29] (see Supplementary Materials S1).

#### 2.1.2. Anthropometrics

All anthropometrics were measured by trained research assistants. Students’ height and weight were measured using portable Seca stadiometers and Seca flat scales. Students’ waist and hip circumference were measured using Seca flexible tape measures. The anthropometric measurements used in this study are BMI (body mass index (kg/m^2^)), waist circumference (cm), waist to hip ratio, and waist to height ratio.

#### 2.1.3. Friendship Nominations

All students were asked “Please rank your top 5 male and top 5 female friends at ASU (the first being your best friend, the second being your next closest friend, and so on).” [37,38,39,40]. Students typed the names of their friends into free-flow text boxes. Information on friends’ residence location was also obtained to ensure that students’ friends were linked to the correct person.

#### 2.1.4. Diet

Meal consumption. Similar to the widely used Project EAT study questionnaire [41], all students were asked “In the past 7 days, how often did you eat the following”: “Breakfast?” [42], “Evening meal?” [43], “ Fast foods (e.g., McDonald’s, Raising Canes, Taco Bell, Dominos, Panda Express, etc.)?” [42], “Sit-down restaurant food (e.g., Olive Garden, Oreganos, etc.)?” [44], “Dining hall food?” [44], “ Home-cooked foods (e.g., meals made from scratch)?” [44]. Response options were in one day increments between “Never” and “7 days”. Test–retest values for breakfast, fast food, and home cooked meal consumption were available from a smaller pilot study conducted among first-year students from the same university the prior year; test–retest values were between 0.58 (home cooked meals) and 0.79 (breakfast) [45].

Dietary Screener Questionnaire (DSQ) computed variables. The Dietary Screener Questionnaire used in the 2009–2010 NHANES, with the two additional questions for sport and energy drinks as per the National Health Interview Survey Cancer Control Supplement 2010 was used to examine dietary intake [46,47]. The NHANES DSQ scoring algorithm has been shown to produce estimates similar to multiple 24-h recalls for those between 2 and 69 years of age [48].

Using the responses from the DSQ, estimated mean intakes of fiber, calcium, whole grains, total added sugars, dairy, fruit and vegetables (including legumes and French fries), vegetables (including legumes and French fries), fruit and vegetables (including legumes and excluding French fries), vegetables (including legumes and excluding French fries), fruits, and added sugars from sugar sweetened beverages (SSBs), were calculated. As recommended, items were recoded to daily frequency.

#### 2.1.5. Physical Activity

The Godin–Shephard questionnaire was used to determine students’ physical activity levels. The widely used Godin–Shephard questionnaire has been shown to provide reasonable physical activity level estimates for children and adults [49,50]. The amount of moderate to vigorous physical activity students engaged in each week was determined by averaging the amount of time students reported engaged in strenuous or moderate exercise in a usual week [51]. Students were also asked “Yesterday, how much time did you spend in front of a screen (excluding time in class and being physically active)? This includes computers, tablets, smartphones, TV, video games, movies, etc.”; this question was validated on a college student population [52]. Response options for strenuous PA, moderate PA, and screen time were “None”, “Less than ½ h”, “½–2 h”, “2 ½–4 h”, “4 ½–6 h”, “More than 6 h,” which were coded as 0, 30, 75, 195, 315 and 360 min respectively.

#### 2.1.6. Alcohol Intake

Studies indicate that self-report measures of alcohol are both reliable and valid [53,54,55,56]. To examine students’ alcohol use, all students were first asked “Have you ever drank alcohol?”. Response options were Yes and No. Students who reported alcohol consumption were asked “For each day of the week in the calendar below, indicate the number of alcoholic drinks typically consumed on that day.” [57]. The number of alcohol drinks reported throughout the week was summed to create an estimated weekly alcohol intake. To examine binge drinking, female students were asked “During the last two weeks, how many times have you had four alcoholic drinks in a row?”; male students were asked “During the last two weeks, how many times have you had five alcoholic drinks in a row?” [58]. Response options were in one day increments from “Never” to “4 or more days”. Students were classified as binge drinking if they reported consuming 4 (females) or 5 (males) alcoholic drinks in a row on at least one day. A test–retest value of 0.66 was obtained from first-year college students from the same university for the same binge drinking question the prior year [45].

#### 2.1.7. Sleep Behaviors

To examine students’ sleep behaviors, all students were asked the following questions adapted from ACHA [59]; adaptations of these questions were validated against diaries and actigraphy in the School Sleep Habits Survey [60]. “In the past 7 days, how often did the following occur?” “You got enough sleep so that you felt rested when you woke up in the morning”, “You woke up too early in the morning and couldn’t get back to sleep”, “You felt tired, dragged out, or sleepy during the day”. Response options were in one day increments between “0 days” and “7 days” and were valued between 0 and 7, respectively.

To determine the number of hours students slept during the weekdays, and weekends, students were asked two questions developed for this current study “On an average weekday, how many hours of sleep do you usually get?”, and “On an average weekend day how many hours of sleep do you usually get?”. Response options were in half hour increments from one to 16 h.

#### 2.1.8. Mental Health

Students’ stress and depressed mood were determined using adapted versions of the Cohen stress measure [61] and the 2013 American College Health Association depression level module [59], as used previously [62]. Both the stress and depressed mood questions have been validated for populations similar to the first-year college students used in this study [61,63]. The test–retest values obtained from a smaller study conducted on first-year college students at the same university the prior year were 0.74 for stress, and 0.89 for depression [45]. Higher scores indicated higher levels of stress and depressed mood.

#### 2.1.9. Food Security Status

To determine students’ food security status, the USDA food security six-item module with a one month time frame was used [64]; two affirmative responses indicated food insecurity. The USDA food security six-item module has been validated against the standard 18-item household food security scale [65].

#### 2.1.10. Demographics

Students’ gender (male/female), race/ethnicity (Non-Hispanic White, Non-Hispanic Black, Hispanic, Other), Pell Grant status (recipient vs. not), year in college (first-year student vs. not), and place of residence were obtained.

## 3. Statistical Analyses

Differences in Key Demographics between the Students Included at the Start and End of the Spring Semester Were Examined Using T- and Chi-Squared Tests as Appropriate.

### 3.1. Are Friends Similar on the Candidate Mediators?

A linear mixed effect model was used to determine if students’ survey responses could be predicted by their friends’ survey responses. Students’ survey responses were predicted by the average of their friends’ survey responses. As students may be more similar at the end, rather than the start, of the semester, a control for whether the survey was completed at the start or end of the semester was included in the model. As students from the same residence hall may be more similar, a control for the students’ residence hall was included in the model. A random effect for students was also included in the model to control for multiple responses (start and end of the semester) by some students.

### 3.2. Are Students’ Anthropometrics Associated with the Candidate Mediators?

A linear mixed effect model was used to determine if the hypothesized mediators predicted students’ anthropometrics (BMI, waist circumference, waist to hip ratio, and waist to height ratio). As students’ anthropometrics may be different at the start and end of the semester, and as students’ anthropometrics may be associated with their residence hall, controls for whether the survey was completed at the start or end of the semester and the students’ residence hall were included in the model. As in the previous analyses, a random effect for students was also included in the models.

### 3.3. Are Changes in Students’ Body Size Associated with the Candidate Mediators?

A linear mixed effect model was then used to determine if changes in students’ body size measurements (Fall 2015 semester start to Spring 2016 semester) were associated with candidate mediators. Students’ anthropometrics in Spring were predicted by the students’ survey response in Spring, after controlling for the students’ anthropometrics at the start of Fall. As there was an extended recruitment period at the start of Fall, and students’ anthropometrics may have been higher at the end of the recruitment period, the date anthropometrics obtained in Fall were included in the models. Controls for whether the Spring survey was completed at the start or end of the Spring semester, students’ residence hall, and a random effect by student were included in the models. By examining the association between students’ survey responses, anthropometrics, and anthropometrics change, the candidate mediators are tested for their potential. If no association exists between the candidate mediator and anthropometrics, it is unlikely that friends influence anthropometrics through the mediator. As such, controls for friends’ anthropometrics and friends’ survey responses were not included in this model.

The above analyses examine if there is any association between friends’ responses to the candidate mediators, and if the candidate mediators are associated with anthropometrics. By not controlling for demographics, these analyses simply tested if there was any association at all that would be consistent with mediation. As a second step, the above models were re-run after controlling for students’ sex, race/ethnicity, Pell Grant status, and year in college. These models offer a stricter test of the candidate mediators by evaluating whether the patterns consistent with mediation persist net of controls. A liberal alpha of 0.05 was used to ensure that we did not inadvertently remove any potential mediators that researchers may want to examine further. All analyses were conducted in R (v 3.6.0).

## 4. Results

A total of 509 students were included in the cross-sectional analyses examining friend similarity on the health behaviors (73% female, 46% non-Hispanic White; Table 1). The average number of friendship nominations was 2.69 at the start of the Spring semester, and 2.45 at the end of the Spring semester; on average, the students in this study nominated between two and three friends. A total of 428 students were included in the analyses examining BMI (71% female, 47% non-Hispanic White; Table 2). At the start of the Spring semester, the average BMI was 24.01; the average waist circumference was 81 cm, and the average waist to hip and waist to height ratios were 0.82 and 0.48 respectively. For both samples, the majority of the students (89%, 92%) were in their first year, and 1/3 of the students were Pell Grant recipients.

The bivariate analyses for the cross-sectional analyses examining behavioral similarity among friends indicated that compared to the start of the Spring semester, students reported consuming more dining hall meals and less home cooked meals at the end of the Spring semester (*p* < 0.001; Table 1). These analyses suggested that students were also less likely to report getting enough sleep (*p* < 0.001), were more likely to report being tired throughout the day (*p* < 0.001), reported less hours of sleep on weekdays (*p* = 0.001) and weekends (*p* = 0.006), and reported higher levels of stress (*p* = 0.012) and depression (*p* = 0.014), at the end, rather than the start, of the Spring semester.

The bivariate analyses for the longitudinal analyses examining behavioral predictors of BMI indicated similar changes over the Spring semester (Table 2).

### 4.1. Are Friends Similar on the Candidate Mediators?

The linear mixed effect model for the cross-sectional study indicated that students were similar to their friends in terms of diet, physical activity, alcohol intake, sleep, and mental health. Students’ frequency of consuming breakfast, evening meals, dining hall meals, and home cooked meals was positively associated with their friends’ frequency of consuming these same items (Table 3; model A). Similarly, students’ predicted intakes of fiber, fruits and vegetables (including legumes and including fries, and including legumes and excluding fries), vegetables (including legumes and fries, and including legumes and excluding fries), and fruits were positively associated with their friends’ predicted intakes on the same items. Students’ responses to moderate to vigorous physical activity, ever drinking alcohol, binge drinking, total weekly alcoholic drinks, obtaining enough sleep, waking up too early, hours of sleep on weekdays and weekends, and stress were also positively associated with their friends’ responses to these same items. Similar results were found once demographics were controlled for (Table 3; model B).

### 4.2. Are Students’ Anthropometrics Associated with the Candidate Mediators?

Students’ BMI in the Spring semester was associated with number of restaurant meals, and hours of sleep on weekdays when demographics were included, and excluded in the model (Table 4; BMI in Spring, models A and B). Students’ waist circumference, and waist to height ratio in the Spring semester were associated with calcium and dairy when demographics were included in the model (Table 5 and Table 6; waist circumference in the Spring semester and waist to height ratio in Spring, model A). No other statistically significant associations between the variables tested and students’ anthropometrics in the Spring semester were observed.

### 4.3. Are Changes in Students’ Body Size Associated with the Candidate Mediators?

Students’ anthropometric change from the start of Fall 2015 to Spring 2016 was also not associated with most aspects of students’ reported diet, physical activity, alcohol intake, sleep, mental health, or food security status in Spring 2016 when demographics were excluded, and included, in the models (Table 4, Table 5, Table 6 and Table 7; anthropometric change from start of Fall to Spring, model A and model B). However, use of the dining hall was associated with an increase in BMI, waist circumference, and waist to hip ratio when demographics were excluded from the model; the association between dining hall use and BMI and waist to hip ratio remained significant once demographics were controlled for. Calcium and dairy were associated with all four of the anthropometric measures examined when demographics were excluded from the model; some significant associations remained once demographics were included in the model. Stress and depression were negatively associated with BMI change when demographics were included, and excluded from the models.

## 5. Discussion

Prior work has shown that friends potentially influence each other’s BMI even after taking into account friendship selection and shared environment [27,28,29,30]. This study examined a comprehensive list of weight-related and behavioral mediators suggested by the literature as causal mechanisms underlying this influence. We used validated self-reported measures to assess the possibility of convergence of diet, physical activity, alcohol intake, and sleep behaviors, as well as mental health and food insecurity among friends, and their impact on anthropometrics. Despite using measured anthropometrics and a wider and stronger set of behavioral measures, the majority of the variables examined were not convincing candidates that might account for the increasing convergence in anthropometrics among friends observed in this sample, similarly to Madan et al. [34]. Friends were similar on many hypothesized mediators, but the mediator candidates that friends were similar on were not often associated with anthropometrics or anthropometric change, and hence cannot be responsible for observed changes in BMI or other anthropometrics in the current study. This study examines the suitability of the hypothesized mediators for explaining why friends’ anthropometrics tend to become more similar over time. Dining hall meal frequency, and stress show the most suitability for further study. However, given the large number of tests, the observed associations with even these few variables may be spurious.

To date, the majority of the mediators that we, and others [34], have assessed have not had similar values among friends and been significantly associated with anthropometric change. We suggest that there are four potential reasons for this finding. First, it is possible that the self-reported measures used in this study (though previously validated) were not sensitive, valid, or reliable enough to capture the mediating role between friends’ weight-related behaviors and weight change. Given the known limitations of self-reported data [66], particularly for diet [67], more sensitive measures such as multiple 24-h recalls may be necessary to observe effects on weight change. In population-level studies, particularly those with over 1000 participants, self-reported measures are often required given the expense of objective measures such as meal observations and accelerometry. Nonetheless, if feasible, objective measures would be ideal. Other methods of collecting data, such as ecological momentary assessments (EMAs), may also help mitigate some of the limitations with traditional self-reported data [68,69,70]. EMAs collect data in real-time and virtually eliminate recall bias historically associated with self-reported diet and physical activity (including alcohol intake and sleep), and emotional wellbeing involving longer-term recall. Second, weight gain may be a result of specific interactions between these, or other, measures. Specific combinations of behaviors, rather than isolated behaviors, may serve as the basis for influence on weight gain or loss [71]. Third, other domains that we did not consider as potential mediators may explain the effect of friends on college students’ anthropometrics over time. For example, Madan et al. suggested that it was not friends themselves, but proximity to peers with certain behaviors and weight status that explained the convergence of BMI among peers over time [34], suggesting a broader set of influential peers than close friends. Alternatively, emerging science suggests that meal times and circadian rhythms are associated with obesity [72,73,74]. College students’ engagement with friends at meals and known lack of aberrance to circadian rhythms, may be some other factors to consider as mediators. Fourth, while this study examined anthropometric change over one academic year, longer periods of time may be needed to obtain reliable estimates of associations between such factors and weight gain.

While the majority of candidate mediators examined in this study could not explain why friends tend to have more similar BMIs over time, several other results were found. Compared to the start of the Spring semester, students reported consuming more dining hall meals, and less home cooked meals, at the end of the Spring semester. The magnitude of the changes suggests that students replace approximately one home cooked meal with one dining hall meal between the start and end of the Spring semester. The reason for the change in meals over the Spring semester was unclear. One explanation may be that students have basic meal ingredients provided to them by parents/caregivers at the start of the semester that run out by the end of the semester; students may turn to the dining halls once their alternative food sources run out. Changes in sleep behaviors were also found, with students reporting poorer sleep behaviors at the end, rather than the start, of the Spring semester. Prior studies have reported an association between stress and depression and poor sleep [75,76]; students stress and depression levels were higher at the end, rather than the start, of the Spring semester. Students typically completed the two waves of Spring surveys in January and April, respectively, such that the survey at the end of the Spring semester was within one month of final exams (final exams were during the first week of May). Future studies should examine why students’ source of meals, sleep behaviors, and stress and depression levels changed between the start and end of the semester.

This study, like several prior studies, found that friends were similar on many dietary variables [32,33,34], physical activity [28,33,77,78], alcohol intake [79,80,81], and sleep [82]. Prior findings also suggested that adolescents influence each other’s’ depression levels [83,84]; while the current study did not find a link among friends for depression, we found an association for stress among nominated friends. Reports of stress in college, particularly first-year students, are common [85,86,87]. In addition, first generation and students of color have higher reported levels of stress [88,89,90,91,92,93,94,95]; this study sample had relatively high participation among both groups. Research indicates that social support is one of the strongest buffers against stress during this critical time period [96,97,98]. Friends can play an important role of decreasing stress among emerging adults, but they may also perpetuate the effects of stress if they have poor coping skills themselves. Given the known effects of stress on cardiometabolic outcomes [99,100], future research should explore the mechanisms on how diverse students experience stress together, and how stress impacts health outcomes such as weight status.

## 6. Study Strengths and Limitations

The strengths of this study are the large number of college students, the longitudinal design, the objective measures of anthropometrics, and the use of validated measures for diet, physical activity, alcohol intake, sleep, mental health, and food insecurity. The limitations of this study are the number of students who were lost to follow up and the incomplete friendship network. The number of friendships captured by this study were low, with around 1/3 of students having only one friend in the study (Start of Spring: 32%, End of Spring: 36%); many students were excluded from the analysis examining friend similarity due to none of their friends being captured in this study. Students likely had other friends, not captured in this study, who also influenced their behaviors. This study also only examined the friendship network within the university; friendships outside of the university setting may also have influenced students’ health. We examined a large number of potential mediators and their relationship with several outcomes. Given the hundreds of tests in this study, many of the specific associations are likely spurious. Even when casting a broad net, we only identify two potential mediators. This suggests that future work needs to (1) carefully scrutinize the potential of these two mediators, (2) explore alternative forms of measurement for the full range of mediators, and (3) explore other alternative mediators. Finally, students may have been influenced by people they did not consider to be “friends,” such as their roommate or their resident assistant.

## 7. Conclusions

Dining hall use, hours of sleep on weekdays, and stress emerged as potential candidate mediators for the relationship between friends’ and college students’ anthropometrics. We did not find strong evidence that any of the other measures examined in this study explained the similarity of friends’ anthropometrics. Future research should use social network analyses to explore the relationship of friendship selection and friendship influence on dining hall use, hours of sleep on weekdays, and stress and how these impact the anthropometrics of emerging adults. More research is needed using even stronger measures of self-report or objective assessments to confirm or disprove these findings. Alternative means of assessing these measures such as EMAs are also warranted means to examine these findings further. Future research should also consider additional candidates for the purpose of mediation.

## Figures and Tables

**Table 1:** Key demographics of students included in cross-sectional analyses examining the association of friend similarity with weight-related behavioral factors (*n* = 509).

Variable	Start of Spring	End of Spring	*p*-Value
*N*	*n* = 432	*n* = 358	
Demographics, n (%)			
Sex			1.000
Female	318 (73.6)	263 (73.5)	
Male	114 (26.4)	95 (26.5)	
Race/Ethnicity			0.966
Non-Hispanic White	203 (47.0)	163 (45.5)	
Non-Hispanic Black	43 (10.0)	39 (10.9)	
Hispanic	113 (26.2)	95 (26.5)	
Other	73 (16.9)	61 (17.0)	
Pell Grant Recipient			0.778
No	284 (65.7)	231 (64.5)	
Yes	148 (34.3)	127 (35.5)	
First-year student			0.403
No	45 (10.4)	45 (12.6)	
Yes	387 (89.6)	313 (87.4)	
Residence Hall			0.854
A	288 (66.7)	227 (63.4)	
B	28 (6.5)	22 (6.1)	
C	55 (12.7)	50 (14.0)	
D	13 (3.0)	16 (4.5)	
E	24 (5.6)	23 (6.4)	
F	24 (5.6)	20 (5.6)	
Friendship nominations, mean (SD)	2.7 (1.6)	2.5 (1.5)	0.037
Diet, mean (SD)			
Meal consumption			
Breakfast	4.1 (2.4)	3.8 (2.3)	0.122
Evening meals	6.0 (1.5)	5.9 (1.6)	0.326
Fast food	1.7 (1.6)	1.8 (1.5)	0.536
Restaurant meals	1.3 (1.3)	1.2 (1.5)	0.315
Dining hall meals	3.8 (2.3)	5.1 (1.9)	**<0.001**
Home cooked meals	2.1 (2.1)	1.1 (1.6)	**<0.001**
Diet screener Questionnaire computed variables			
Fiber (g/day)	15.2 (3.2)	15.0 (3.1)	0.276
Calcium (mg/day)	950 (219)	958 (232)	0.609
Whole grains (oz/day)	0.7 (0.3)	0.7 (0.3)	0.940
Total added sugars (tsp/day)	16.8 (7.4)	16.7 (6.8)	0.808
Dairy (cups/day)	1.6 (0.6)	1.6 (0.7)	0.472
F/V (incl legumes and fries; cups/day)	2.4 (0.7)	2.3 (0.6)	0.065
Vegetables (incl legumes and fries; cups/day)	1.5 (0.3)	1.4 (0.3)	0.197
F/V (incl legumes, excl fries; cups/day)	2.2 (0.7)	2.2 (0.7)	0.160
Vegetables (incl legumes and fries; cups/day)	1.3 (0.4)	1.3 (0.3)	0.124
Fruits (cups/day)	0.9 (0.5)	0.9 (0.5)	0.621
Added sugars from SSB (tsp/day)	8.0 (6.3)	8.0 (5.9)	0.972
Physical activity, mean (SD)			
Moderate–Vigorous PA (mins/day)	42.4 (31.2)	42.2 (31.4)	0.922
Screen Time (mins/day)	203 (108)	205 (120)	0.754
Alcohol intake			
Ever drunk alcohol, n (%)	279 (65.3)	215 (60.9)	0.229
Binge drinking, n (%)	116 (27.3)	76 (21.6)	0.080
Total weekly drinks, mean (SD)	2.9 (5.8)	2.6 (4.8)	0.336
Sleep behaviors, mean (SD)			
Enough sleep	3.6 (2.0)	2.9 (2.0)	**<0.001**
Woke up too early	1.8 (2.0)	1.9 (2.0)	0.558
Tired during day	3.3 (2.1)	4.0 (2.1)	**<0.001**
Hours of sleep on weekdays	7.2 (1.3)	6.8 (1.5)	**0.001**
Hours of sleep on weekends	8.5 (1.7)	8.2 (1.9)	**0.006**
Mental Health, mean (SD)			
Stress	8.1 (2.4)	8.5 (2.5)	**0.012**
Depression	2.0 (0.7)	2.1 (0.8)	**0.014**
Food Security status, n (%)			
Food insecure	135 (31.7)	116 (32.9)	0.786

F/V: Fruits and vegetables; SSB: Sugar Sweetened Beverages; PA: Physical Activity. Bolded text indicates statistical significance.

**Table 2:** Key demographics of students included in the longitudinal analyses examining the association of weight-related behavioral factors with BMI over time (*n* = 428).

Variable	Start of Spring	End of Spring	*p*-Value
*N*	*n* = 383	*n* = 335	
Demographics, n (%)			
Sex			0.736
Female	273 (71.3)	234 (69.9)	
Male	110 (28.7)	101 (30.1)	
Race/Ethnicity			0.969
Non-Hispanic White	179 (46.7)	156 (46.6)	
Non-Hispanic Black	42 (11.0)	38 (11.3)	
Hispanic	91 (23.8)	83 (24.8)	
Other	71 (18.5)	58 (17.3)	
Pell Grant Recipient			0.786
No	254 (66.3)	218 (65.1)	
Yes	129 (33.7)	117 (34.9)	
First-year student			0.675
No	31 (8.1)	31 (9.3)	
Yes	352 (91.9)	304 (90.7)	
Residence Hall			0.997
A	227 (59.3)	195 (58.2)	
B	21 (5.5)	19 (5.7)	
C	50 (13.1)	47 (14.0)	
D	13 (3.4)	10 (3.0)	
E	37 (9.7)	34 (10.1)	
F	35 (9.1)	30 (9.0)	
Anthropometrics, mean (SD)			
BMI (kg/m^2^)	24.0 (4.2)	24.3 (4.2)	0.439
Waist circumference (cm)	81.1 (11.2)	81.3 (11.1)	0.795
Waist to hip ratio	0.8 (0.1)	0.8 (0.1)	0.484
Waist to height ratio	0.5 (0.1)	0.5 (0.1)	0.929
Diet, mean (SD)			
Meal consumption			
Breakfast	4.1 (2.4)	3.7 (2.4)	**0.030**
Evening meals	6.0 (1.5)	5.8 (1.7)	0.076
Fast food	1.7 (1.7)	1.6 (1.4)	0.388
Restaurant meals	1.3 (1.4)	1.1 (1.3)	**0.014**
Dining hall meals	3.8 (2.3)	5.0 (1.9)	**<0.001**
Home cooked meals	2.3 (2.2)	1.1 (1.6)	**<0.001**
Diet screener Questionnaire computed variables			
Fiber (g/day)	15.2 (3.2)	15.1 (3.2)	0.786
Calcium (mg/day)	946 (218)	963 (239)	0.345
Whole grains (oz/day)	0.7 (0.3)	0.7 (0.3)	0.896
Total added sugars (tsp/day)	16.3 (6.7)	16.9 (7.3)	0.251
Dairy (cups/day)	1.6 (0.6)	1.6 (0.6)	0.524
F/V (incl legumes and fries; cups/day)	2.3 (0.6)	2.3 (0.7)	0.742
Vegetables (incl legumes and fries; cups/day)	1.5 (0.3)	1.5 (0.3)	0.869
F/V (incl legumes, excl fries; cups/day)	2.2 (0.7)	2.2 (0.7)	0.950
Vegetables (incl legumes and fries; cups/day)	1.3 (0.3)	1.3 (0.4)	0.911
Fruits (cups/day)	0.9 (0.4)	0.9 (0.5)	0.600
Added sugars from SSB (tsp/day)	7.9 (6.3)	8.2 (6.2)	0.469
Physical activity, mean (SD)			
Moderate–Vigorous PA (mins/day)	40.6 (30.7)	40.5 (30.7)	0.965
Screen Time (mins/day)	203 (108)	216 (117)	0.106
Alcohol intake			
Ever drunk alcohol, n (%)	226 (59.6)	193 (57.8)	0.672
Binge drinking, n (%)	93 (24.7)	67 (20.1)	0.169
Total weekly drinks, mean (SD)	2.8 (6.1)	2.7 (5.8)	0.811
Sleep behaviors, mean (SD)			
Enough sleep	3.6 (2.0)	2.9 (1.9)	**<0.001**
Woke up too early	1.9 (2.0)	1.7 (1.9)	0.325
Tired during day	3.3 (2.1)	3.9 (2.1)	**<0.001**
Hours of sleep on weekdays	7.1 (1.4)	6.9 (1.5)	**0.046**
Hours of sleep on weekends	8.5 (1.8)	8.2 (1.8)	**0.028**
Mental Health, mean (SD)			
Stress	8.1 (2.5)	8.5 (2.5)	**0.020**
Depression	2.0 (0.8)	2.2 (0.8)	**0.014**
Food Security status, n (%)			
Food insecure	116 (30.7)	115 (34.4)	0.325

F/V: Fruits and vegetables; SSB: Sugar Sweetened Beverages; PA: Physical Activity. Bolded text indicates statistical significance.

**Table 3:** Results of the cross-sectional linear mixed effect model examining the similarity between students’ and their friends’ weight-related behavioral survey responses.

Variable			Model A		Model B	
	npid	nresp	β	95% CI	β	95% CI
Diet						
Meal consumption						
Breakfast	493	766	**0.11**	**(0.02, 0.19)**	**0.11**	**(0.02, 0.19)**
Evening meals	492	765	**0.12**	**(0.03, 0.20)**	**0.11**	**(0.03, 0.20)**
Fast food	493	766	0.07	(−0.02, 0.17)	0.07	(−0.03, 0.16)
Restaurant meals	493	766	0.07	(−0.03, 0.16)	0.06	(−0.04, 0.15)
Dining hall meals	493	766	**0.19**	**(0.10, 0.28)**	**0.16**	**(0.07, 0.25)**
Home cooked meals	492	763	**0.17**	**(0.08, 0.26)**	**0.16**	**(0.07, 0.25)**
Diet screener Questionnaire computed variables						
Fiber (g/day)	490	753	**0.13**	**(0.04, 0.22)**	0.04	(−0.05, 0.13)
Calcium (mg/day) ^A^	490	753	0.04	(−0.04, 0.13)	0.00	(−0.08, 0.07)
Whole grains (oz/day)	493	760	−0.06	(−0.15, 0.04)	−0.07	(−0.17, 0.02)
Total added sugars (tsp/day)	491	760	0.07	(−0.02, 0.16)	0.05	(−0.04, 0.14)
Dairy (cups/day)	493	762	0.01	(−0.08, 0.10)	0.00	(−0.08, 0.08)
F/V (incl legumes and fries; cups/day)	492	757	**0.16**	**(0.07, 0.25)**	**0.13**	**(0.04, 0.22)**
Vegetables (incl legumes and fries; cups/day)	492	757	**0.14**	**(0.05, 0.23)**	0.08	(−0.01, 0.17)
F/V (incl legumes, excl fries; cups/day)	492	757	**0.14**	**(0.05, 0.23)**	**0.12**	**(0.03, 0.21)**
Vegetables (incl legumes and fries; cups/day)	492	757	**0.12**	**(0.03, 0.21)**	0.07	(−0.02, 0.16)
Fruits (cups/day)	494	766	**0.13**	**(0.05, 0.22)**	**0.13**	**(0.04, 0.22)**
Added sugars from SSB (tsp/day)	492	764	0.05	(−0.04, 0.14)	0.04	(−0.05, 0.13)
Physical activity						
Moderate–Vigorous PA (mins/day) ^A^	491	760	**0.10**	**(0.01, 0.19)**	0.08	(−0.01, 0.17)
Screen Time (mins/day) ^A^	491	760	−0.01	(−0.10, 0.08)	−0.01	(−0.10, 0.08)
Alcohol intake						
Ever drunk alcohol, n (%)	495	769	**0.26**	**(0.18, 0.35)**	**0.25**	**(0.17, 0.34)**
Binge drinking, n (%)	492	764	**0.23**	**(0.14, 0.32)**	**0.21**	**(0.12, 0.30)**
Total weekly drinks	495	769	**0.25**	**(0.19, 0.31)**	**0.24**	**(0.18, 0.31)**
Sleep behaviors						
Enough sleep	491	760	**0.12**	**(0.02, 0.21)**	**0.11**	**(0.02, 0.20)**
Woke up too early	491	760	**0.13**	**(0.05, 0.22)**	**0.13**	**(0.04, 0.22)**
Tired during day	491	760	0.08	(−0.02, 0.17)	0.07	(−0.02, 0.16)
Hours of sleep on weekdays	494	765	**0.10**	**(0.01, 0.20)**	**0.10**	**(0.00, 0.19)**
Hours of sleep on weekends	493	764	**0.19**	**(0.10, 0.28)**	**0.19**	**(0.10, 0.28)**
Mental Health						
Stress	495	769	**0.10**	**(0.00, 0.19)**	**0.10**	**(0.00, 0.19)**
Depression	495	769	0.08	(−0.01, 0.17)	0.07	(−0.01, 0.16)
Food Security status						
Food insecure	495	769	0.02	(−0.07, 0.11)	0.01	(−0.09, 0.10)

^A^ The variable values were divided by 100 to scale up the beta co-efficient. Model A does not control for student demographics. Model B controlled for students’ sex, race/ethnicity, Pell Grant status, and year in college. F/V: Fruits and vegetables; SSB: Sugar Sweetened Beverages; PA: Physical Activity; npid: number of participants; nresp: number of responses. Bolded text indicates statistical significance.

**Table 4:** Results of the longitudinal linear mixed effect models examining if students’ survey responses on weight-related behavioral factors were associated with their BMI, or BMI change.

Variable			BMI in Spring				BMI Change from Start of Fall to Spring			
			Model A		Model B		Model A		Model B	
	npid	nresp	β	95% CI	β	95% CI	β	95% CI	β	95% CI
Diet										
Meal consumption										
Breakfast	423	710	−0.02	(−0.07, 0.02)	−0.02	(−0.07, 0.02)	0.00	(−0.03, 0.03)	0.00	(−0.03, 0.03)
Evening meals	422	708	0.03	(−0.03, 0.08)	0.03	(−0.02, 0.08)	0.03	(−0.01, 0.07)	0.03	(−0.01, 0.07)
Fast food	423	710	0.01	(−0.04, 0.06)	0.01	(−0.05, 0.06)	0.01	(−0.03, 0.06)	0.01	(−0.03, 0.05)
Restaurant meals	423	710	**0.07**	**(0.01, 0.12)**	**0.07**	**(0.01, 0.13)**	0.02	(−0.03, 0.07)	0.02	(−0.03, 0.07)
Dining hall meals	423	710	0.03	(−0.01, 0.06)	0.02	(−0.01, 0.06)	**0.04**	**(0.01, 0.07)**	**0.04**	**(0.01, 0.07)**
Home cooked meals	423	709	−0.03	(−0.07, 0.01)	−0.03	(−0.07, 0.01)	−0.03	(−0.07, 0.00)	−0.03	(−0.06, 0.00)
Diet screener Questionnaire computed variables										
Fiber (g/day)	418	699	0.01	(−0.02, 0.05)	0.01	(−0.03, 0.05)	0.01	(−0.01, 0.04)	0.01	(−0.02, 0.03)
Calcium (mg/day) ^A^	418	699	0.03	(−0.02, 0.08)	0.02	(−0.03, 0.07)	**0.05**	**(0.01, 0.08)**	**0.05**	**(0.01, 0.09)**
Whole grains (oz/day)	422	706	−0.01	(−0.28, 0.26)	−0.01	(−0.28, 0.27)	0.07	(−0.15, 0.29)	0.06	(−0.16, 0.28)
Total added sugars (tsp/day)	421	706	−0.01	(−0.02, 0.01)	−0.01	(−0.02, 0.01)	0.00	(−0.01, 0.01)	−0.01	(−0.02, 0.01)
Dairy (cups/day)	422	707	0.09	(−0.06, 0.24)	0.09	(−0.06, 0.23)	**0.15**	**(0.04, 0.27)**	**0.15**	**(0.03, 0.27)**
F/V (incl legumes and fries; cups/day)	419	701	−0.02	(−0.17, 0.13)	−0.02	(−0.17, 0.13)	−0.02	(−0.14, 0.09)	−0.04	(−0.15, 0.08)
Vegetables (incl legumes and fries; cups/day)	419	701	0.04	(−0.25, 0.32)	0.03	(−0.26, 0.32)	0.09	(−0.13, 0.31)	0.07	(−0.16, 0.30)
F/V (incl legumes, excl fries; cups/day)	419	702	−0.01	(−0.15, 0.14)	−0.01	(−0.15, 0.14)	−0.01	(−0.12, 0.10)	−0.02	(−0.13, 0.09)
Vegetables (incl legumes and fries; cups/day)	419	702	0.03	(−0.25, 0.30)	0.02	(−0.25, 0.30)	0.07	(−0.14, 0.28)	0.06	(−0.15, 0.28)
Fruits (cups/day)	423	709	−0.07	(−0.27, 0.12)	−0.08	(−0.27, 0.12)	−0.07	(−0.22, 0.08)	−0.08	(−0.23, 0.07)
Added sugars from SSB (tsp/day)	422	708	−0.01	(−0.03, 0.01)	−0.01	(−0.03, 0.01)	−0.01	(−0.02, 0.01)	−0.01	(−0.02, 0.01)
Physical activity, mean (SD)										
Moderate–Vigorous PA (mins/day) ^A^	422	707	−0.02	(−0.33, 0.30)	−0.02	(−0.33, 0.30)	0.01	(−0.23, 0.25)	0.01	(−0.23, 0.26)
Screen Time (mins/day) ^A^	422	708	0.05	(−0.03, 0.12)	0.05	(−0.03, 0.12)	0.04	(−0.02, 0.11)	0.05	(−0.02, 0.11)
Alcohol intake										
Ever drunk alcohol	424	713	−0.10	(−0.36, 0.17)	−0.09	(−0.36, 0.18)	−0.07	(−0.25, 0.10)	−0.08	(−0.25, 0.09)
Binge drinking	423	709	0.11	(−0.12, 0.34)	0.11	(−0.12, 0.34)	0.09	(−0.08, 0.27)	0.08	(−0.10, 0.25)
Total weekly drinks	424	713	0.00	(−0.02, 0.02)	0.00	(−0.02, 0.02)	0.00	(−0.01, 0.02)	0.00	(−0.01, 0.02)
Sleep behaviors, mean (SD)										
Enough sleep	423	707	−0.03	(−0.07, 0.02)	−0.03	(−0.07, 0.02)	0.00	(−0.03, 0.04)	0.00	(−0.04, 0.04)
Woke up too early	423	707	−0.01	(−0.06, 0.03)	−0.01	(−0.06, 0.03)	−0.01	(−0.04, 0.03)	−0.01	(−0.04, 0.03)
Tired during day	423	707	−0.01	(−0.06, 0.03)	−0.01	(−0.05, 0.03)	−0.02	(−0.05, 0.02)	−0.02	(−0.05, 0.02)
Hours of sleep on weekdays	424	713	**−0.07**	**(−0.12, −0.01)**	**−0.06**	**(−0.12, −0.01)**	−0.02	(−0.06, 0.03)	−0.02	(−0.06, 0.03)
Hours of sleep on weekends	424	713	−0.01	(−0.05, 0.04)	−0.01	(−0.05, 0.04)	0.01	(−0.03, 0.05)	0.01	(−0.03, 0.05)
Mental Health, mean (SD)										
Stress	423	712	−0.02	(−0.07, 0.02)	−0.02	(−0.07, 0.02)	**−0.05**	**(−0.08, −0.01)**	**−0.05**	**(−0.08, −0.02)**
Depression	423	712	−0.02	(−0.16, 0.12)	−0.02	(−0.16, 0.12)	**−0.11**	**(−0.21, −0.01)**	**−0.11**	**(−0.21, −0.01)**
Food Security status, n (%)										
Food insecure	423	712	−0.06	(−0.25, 0.14)	−0.06	(−0.26, 0.13)	0.02	(−0.13, 0.17)	0.01	(−0.14, 0.16)

^A^ The variable values were divided by 100 to scale up the beta co-efficient. Model A does not control for student demographics. Model B controlled for students’ sex, race/ethnicity, Pell Grant status, and year in college. F/V: Fruits and vegetables; SSB: Sugar Sweetened Beverages; PA: Physical Activity; npid: number of participants; nresp: number of responses. Bolded text indicates statistical significance.

**Table 5:** Results of the longitudinal linear mixed effect models examining if students’ survey responses on weight-related behavioral factors were associated with their waist circumference, or waist circumference change.

Variable			Waist Circumference in Spring				Waist Circumference Change from Start of Fall to Spring			
			Model A		Model B		Model A		Model B	
	npid	nresp	β	95% CI	β	95% CI	Β	95% CI	β	95% CI
Diet										
Meal consumption										
Breakfast	423	710	−0.14	(−0.35, 0.07)	−0.13	(−0.34, 0.08)	0.00	(−0.14, 0.15)	0.00	(−0.14, 0.14)
Evening meals	422	708	−0.02	(−0.26, 0.22)	−0.03	(−0.27, 0.22)	0.02	(−0.17, 0.21)	0.01	(−0.19, 0.20)
Fast food	423	710	0.03	(−0.22, 0.28)	0.00	(−0.25, 0.25)	0.08	(−0.11, 0.28)	0.07	(−0.13, 0.26)
Restaurant meals	423	710	0.10	(−0.18, 0.38)	0.10	(−0.18, 0.39)	−0.03	(−0.25, 0.20)	−0.02	(−0.25, 0.20)
Dining hall meals	423	710	0.08	(−0.10, 0.26)	0.04	(−0.13, 0.22)	**0.16**	**(0.02, 0.30)**	0.13	(−0.01, 0.27)
Home cooked meals	423	709	−0.13	(−0.32, 0.06)	−0.11	(−0.30, 0.09)	−0.06	(−0.22, 0.09)	−0.04	(−0.20, 0.11)
Diet screener Questionnaire computed variables										
Fiber (g/day)	418	699	0.09	(−0.08, 0.25)	−0.02	(−0.19, 0.15)	0.03	(−0.08, 0.14)	−0.04	(−0.16, 0.08)
Calcium (mg/day) ^A^	418	699	**0.29**	**(0.06, 0.51)**	0.10	(−0.14, 0.34)	**0.26**	**(0.11, 0.41)**	**0.20**	**(0.03, 0.38)**
Whole grains (oz/day)	422	706	−0.07	(−1.36, 1.22)	−0.27	(−1.55, 1.02)	0.22	(−0.76, 1.20)	0.06	(−0.93, 1.05)
Total added sugars (tsp/day)	421	706	−0.01	(−0.08, 0.05)	−0.03	(−0.10, 0.03)	0.02	(−0.03, 0.06)	0.00	(−0.04, 0.05)
Dairy (cups/day)	422	707	**0.72**	**(0.03, 1.41)**	0.41	(−0.29, 1.10)	**0.95**	**(0.44, 1.45)**	**0.79**	**(0.25, 1.33)**
F/V (incl legumes and fries; cups/day)	419	701	0.05	(−0.66, 0.76)	−0.19	(−0.90, 0.52)	0.03	(−0.47, 0.54)	−0.15	(−0.67, 0.37)
Vegetables (incl legumes and fries; cups/day)	419	701	0.31	(−1.03, 1.66)	−0.25	(−1.61, 1.11)	0.26	(−0.72, 1.24)	−0.15	(−1.17, 0.88)
F/V (incl legumes, excl fries; cups/day)	419	702	0.00	(−0.67, 0.68)	−0.18	(−0.85, 0.49)	0.01	(−0.47, 0.5)	−0.13	(−0.62, 0.36)
Vegetables (incl legumes and fries; cups/day)	419	702	0.23	(−1.04, 1.51)	−0.19	(−1.47, 1.10)	0.20	(−0.74, 1.14)	−0.12	(−1.08, 0.85)
Fruits (cups/day)	423	709	−0.34	(−1.24, 0.57)	−0.48	(−1.39, 0.43)	−0.09	(−0.77, 0.59)	−0.22	(−0.91, 0.46)
Added sugars from SSB (tsp/day)	422	708	−0.01	(−0.09, 0.06)	−0.03	(−0.10, 0.05)	0.02	(−0.04, 0.07)	0.01	(−0.05, 0.06)
Physical activity, mean (SD)										
Moderate–Vigorous PA (mins/day) ^A^	422	707	−0.61	(−2.08, 0.86)	−0.78	(−2.24, 0.69)	−0.35	(−1.42, 0.72)	−0.52	(−1.60, 0.55)
Screen Time (mins/day) ^A^	422	708	0.01	(−0.35, 0.37)	0.01	(−0.35, 0.38)	0.07	(−0.21, 0.35)	0.08	(−0.20, 0.36)
Alcohol intake										
Ever drunk alcohol	424	713	0.00	(−1.17, 1.18)	−0.10	(−1.27, 1.08)	−0.24	(−0.97, 0.49)	−0.34	(−1.08, 0.40)
Binge drinking	423	709	−0.26	(−1.32, 0.79)	−0.39	(−1.44, 0.66)	−0.08	(−0.85, 0.69)	−0.12	(−0.89, 0.65)
Total weekly drinks	424	713	0.00	(−0.09, 0.10)	−0.01	(−0.11, 0.08)	0.01	(−0.05, 0.07)	0.00	(−0.06, 0.06)
Sleep behaviors										
Enough sleep	423	707	0.08	(−0.13, 0.29)	0.06	(−0.15, 0.27)	0.13	(−0.03, 0.29)	0.10	(−0.06, 0.26)
Woke up too early	423	707	−0.03	(−0.23, 0.17)	−0.02	(−0.22, 0.18)	0.00	(−0.15, 0.16)	0.01	(−0.14, 0.17)
Tired during day	423	707	−0.03	(−0.22, 0.16)	−0.02	(−0.22, 0.17)	−0.07	(−0.22, 0.08)	−0.06	(−0.20, 0.09)
Hours of sleep on weekdays	424	713	0.00	(−0.28, 0.28)	0.02	(−0.25, 0.30)	0.05	(−0.16, 0.27)	0.05	(−0.16, 0.27)
Hours of sleep on weekends	424	713	0.08	(−0.14, 0.30)	0.08	(−0.14, 0.30)	0.01	(−0.16, 0.18)	0.00	(−0.17, 0.17)
Mental Health										
Stress	423	712	0.03	(−0.17, 0.22)	0.04	(−0.15, 0.24)	−0.10	(−0.24, 0.03)	−0.08	(−0.22, 0.05)
Depression	423	712	−0.26	(−0.90, 0.38)	−0.18	(−0.82, 0.46)	−0.42	(−0.86, 0.01)	−0.37	(−0.80, 0.07)
Food Security status, n (%)										
Food insecure	423	712	−0.20	(−1.12, 0.72)	−0.29	(−1.20, 0.62)	0.00	(−0.68, 0.69)	−0.03	(−0.71, 0.65)

^A^ The variable values were divided by 100 to scale up the beta co-efficient. Model A does not control for student demographics. Model B controlled for students’ sex, race/ethnicity, Pell Grant status, and year in college. F/V: Fruits and vegetables; SSB: Sugar Sweetened Beverages; PA: Physical Activity; npid: number of participants; nresp: number of responses. Bolded text indicates statistical significance.

**Table 6:** Results of the longitudinal linear mixed effect models examining if students’ survey responses on weight-related behavioral factors were associated with their waist to hip ratio, or waist to hip ratio change ^B^.

Variable			Waist to Hip Ratio in Spring				Waist to hip Ratio Change from Start of Fall to Spring			
			Model A		Model B		Model A		Model B	
	npid	nresp	β	95% CI	β	95% CI	β	95% CI	β	95% CI
Diet										
Meal consumption										
Breakfast	423	710	−0.08	(−0.25, 0.09)	−0.07	(−0.23, 0.10)	0.00	(−0.13, 0.13)	−0.01	(−0.15, 0.12)
Evening meals	422	708	0.14	(−0.07, 0.36)	0.12	(−0.09, 0.32)	0.14	(−0.04, 0.32)	0.11	(−0.07, 0.29)
Fast food	423	710	−0.11	(−0.33, 0.10)	−0.15	(−0.36, 0.06)	−0.10	(−0.28, 0.09)	−0.10	(−0.29, 0.08)
Restaurant meals	423	710	−0.07	(−0.31, 0.18)	−0.07	(−0.31, 0.17)	−0.16	(−0.36, 0.05)	−0.17	(−0.38, 0.04)
Dining hall meals	423	710	0.15	(0.00, 0.30)	0.08	(−0.07, 0.24)	**0.18**	**(0.05, 0.31)**	**0.14**	**(0.01, 0.28)**
Home cooked meals	423	709	−0.08	(−0.25, 0.09)	−0.05	(−0.22, 0.11)	−0.09	(−0.24, 0.06)	−0.08	(−0.22, 0.07)
Diet screener Questionnaire computed variables										
Fiber (g/day)	418	699	0.08	(−0.05, 0.21)	−0.10	(−0.23, 0.04)	0.01	(−0.09, 0.11)	−0.10	(−0.21, 0.01)
Calcium (mg/day) ^A^	418	699	**0.27**	**(0.09, 0.45)**	−0.05	(−0.24, 0.15)	**0.21**	**(0.07, 0.35)**	0.06	(−0.10, 0.23)
Whole grains (oz/day)	422	706	−0.10	(−1.20, 0.99)	−0.51	(−1.59, 0.56)	−0.06	(−0.98, 0.86)	−0.37	(−1.29, 0.55)
Total added sugars (tsp/day)	421	706	0.03	(−0.03, 0.08)	−0.01	(−0.06, 0.05)	0.04	(−0.01, 0.08)	0.02	(−0.02, 0.06)
Dairy (cups/day)	422	707	**0.58**	**(0.01, 1.16)**	−0.01	(−0.58, 0.60)	**0.72**	**(0.25, 1.20)**	0.42	(−0.09, 0.92)
F/V (incl legumes and fries; cups/day)	419	701	0.14	(−0.45, 0.72)	−0.28	(−0.86, 0.30)	0.05	(−0.43, 0.52)	−0.24	(−0.72, 0.25)
Vegetables (incl legumes and fries; cups/day)	419	701	0.49	(−0.63, 1.61)	−0.55	(−1.68, 0.59)	0.06	(−0.86, 0.98)	−0.62	(−1.58, 0.33)
F/V (incl legumes, excl fries; cups/day)	419	702	0.12	(−0.44, 0.67)	−0.22	(−0.77, 0.33)	0.06	(−0.39, 0.51)	−0.17	(−0.63, 0.29)
Vegetables (incl legumes and fries; cups/day)	419	702	0.41	(−0.65, 1.48)	−0.38	(−1.45, 0.69)	0.05	(−0.83, 0.93)	−0.49	(−1.39, 0.41)
Fruits (cups/day)	423	709	-0.27	(−1.04, 0.49)	−0.54	(−1.30, 0.21)	−0.09	(−0.72, 0.55)	−0.28	(−0.92, 0.36)
Added sugars from SSB (tsp/day)	422	708	0.02	(−0.04, 0.08)	0.00	(−0.06, 0.06)	0.03	(−0.01, 0.08)	0.03	(−0.02, 0.08)
Physical activity, mean (SD)										
Moderate–Vigorous PA (mins/day) ^A^	422	707	−0.87	(−2.09, 0.35)	−1.17	(−2.37, 0.03)	−0.66	(−1.66, 0.34)	−0.94	(−1.94, 0.06)
Screen Time (mins/day) ^A^	422	708	0.11	(−0.20, 0.42)	0.09	(−0.21, 0.40)	0.08	(−0.18, 0.34)	0.07	(−0.19, 0.34)
Alcohol intake										
Ever drunk alcohol	424	713	0.02	(−0.87, 0.92)	−0.14	(−1.01, 0.74)	−0.04	(−0.72, 0.64)	−0.18	(−0.86, 0.50)
Binge drinking	423	709	−0.19	(−1.07, 0.69)	−0.41	(−1.27, 0.45)	−0.22	(−0.94, 0.50)	−0.31	(−1.03, 0.41)
Total weekly drinks	424	713	0.03	(−0.04, 0.10)	−0.01	(−0.07, 0.06)	0.01	(−0.04, 0.07)	0.00	(−0.06, 0.05)
Sleep behaviors										
Enough sleep	423	707	−0.04	(−0.22, 0.14)	−0.08	(−0.25, 0.10)	0.01	(−0.14, 0.16)	−0.02	(−0.18, 0.13)
Woke up too early	423	707	−0.02	(−0.19, 0.16)	0.00	(−0.17, 0.17)	−0.02	(−0.17, 0.13)	−0.01	(−0.15, 0.14)
Tired during day	423	707	−0.02	(−0.19, 0.14)	−0.01	(−0.17, 0.15)	−0.03	(−0.17, 0.11)	−0.02	(−0.15, 0.12)
Hours of sleep on weekdays	424	713	−0.02	(−0.26, 0.21)	0.01	(−0.22, 0.24)	−0.03	(−0.23, 0.17)	−0.03	(−0.23, 0.17)
Hours of sleep on weekends	424	713	−0.02	(−0.21, 0.17)	0.00	(−0.19, 0.18)	−0.08	(−0.24, 0.08)	−0.07	(−0.23, 0.09)
Mental Health										
Stress	423	712	−0.03	(−0.18, 0.13)	0.00	(−0.15, 0.15)	−0.05	(−0.18, 0.07)	−0.03	(−0.16, 0.09)
Depression	423	712	−0.33	(−0.85, 0.18)	−0.22	(−0.72, 0.28)	−0.28	(−0.68, 0.13)	−0.20	(−0.60, 0.20)
Food Security status, n (%)										
Food insecure	423	712	−0.09	(−0.86, 0.69)	−0.22	(−0.98, 0.54)	−0.08	(−0.72, 0.56)	−0.11	(−0.75, 0.53)

^A^ The variable values were divided by 100 to scale up the beta co-efficient. ^B^ Waist to hip ratio was multiplied by 100 to scale up the beta co-efficient. Model A does not control for student demographics. Model B controlled for students’ sex, race/ethnicity, Pell Grant status, and year in college. F/V: Fruits and vegetables; SSB: Sugar Sweetened Beverages; PA: Physical Activity; npid: number of participants; nresp: number of responses. Bolded text indicates statistical significance.

**Table 7:** Results of the longitudinal linear mixed effect models examining if students’ survey responses on weight-related behavioral factors were associated with their waist to height ratio, or waist to height ratio change ^B^.

Variable			Waist to Height Ratio in Spring				Waist to Height Ratio Change from Start of Fall to Spring			
			Model A		Model B		Model A		Model B	
	npid	nresp	β	95% CI	β	95% CI	β	95% CI	β	95% CI
Diet										
Meal consumption										
Breakfast	423	710	−0.08	(−0.20, 0.04)	−0.08	(−0.20, 0.04)	0.00	(−0.09, 0.08)	−0.01	(−0.09, 0.08)
Evening meals	422	708	−0.03	(−0.17, 0.12)	−0.02	(−0.17, 0.12)	0.00	(−0.12, 0.11)	0.00	(−0.12, 0.11)
Fast food	423	710	−0.02	(−0.17, 0.13)	−0.02	(−0.17, 0.13)	0.03	(−0.08, 0.15)	0.03	(−0.09, 0.15)
Restaurant meals	423	710	0.05	(−0.12, 0.22)	0.06	(−0.11, 0.23)	−0.02	(−0.15, 0.11)	−0.02	(−0.15, 0.12)
Dining hall meals	423	710	0.02	(−0.08, 0.13)	0.03	(−0.07, 0.14)	0.08	(0.00, 0.17)	0.08	(−0.01, 0.16)
Home cooked meals	423	709	−0.07	(−0.19, 0.04)	−0.08	(−0.19, 0.04)	−0.04	(−0.13, 0.05)	−0.04	(−0.13, 0.06)
Diet screener Questionnaire computed variables										
Fiber (g/day)	418	699	−0.03	(−0.13, 0.06)	−0.03	(−0.13, 0.07)	−0.01	(−0.08, 0.05)	−0.03	(−0.1, 0.04)
Calcium (mg/day) ^A^	418	699	0.02	(−0.12, 0.15)	0.03	(−0.11, 0.18)	**0.11**	**(0.01, 0.20)**	0.11	(0.00, 0.21)
Whole grains (oz/day)	422	706	−0.37	(−1.14, 0.40)	−0.36	(−1.13, 0.41)	−0.02	(−0.61, 0.57)	−0.07	(−0.66, 0.53)
Total added sugars (tsp/day)	421	706	−0.03	(−0.07, 0.01)	−0.03	(−0.07, 0.01)	0.00	(−0.02, 0.03)	0.00	(−0.03, 0.03)
Dairy (cups/day)	422	707	0.16	(−0.25, 0.57)	0.19	(−0.22, 0.61)	**0.45**	**(0.15, 0.76)**	**0.44**	**(0.11, 0.76)**
F/V (incl legumes and fries; cups/day)	419	701	−0.15	(−0.57, 0.27)	−0.13	(−0.56, 0.29)	−0.07	(−0.37, 0.23)	−0.12	(−0.43, 0.19)
Vegetables (incl legumes and fries; cups/day)	419	701	−0.30	(−1.10, 0.50)	−0.26	(−1.07, 0.55)	−0.06	(−0.65, 0.53)	−0.18	(−0.79, 0.44)
F/V (incl legumes, excl fries; cups/day)	419	702	−0.14	(−0.54, 0.26)	−0.13	(−0.53, 0.28)	−0.07	(−0.35, 0.22)	−0.11	(−0.40, 0.19)
Vegetables (incl legumes and fries; cups/day)	419	702	−0.25	(−1.01, 0.51)	−0.21	(−0.98, 0.56)	−0.07	(−0.63, 0.49)	−0.15	(−0.73, 0.43)
Fruits (cups/day)	423	709	−0.29	(−0.83, 0.25)	−0.28	(−0.82, 0.26)	−0.12	(−0.53, 0.29)	−0.15	(−0.56, 0.26)
Added sugars from SSB (tsp/day)	422	708	−0.02	(−0.06, 0.02)	−0.02	(−0.06, 0.02)	0.01	(−0.02, 0.04)	0.00	(−0.03, 0.04)
Physical activity, mean (SD)										
Moderate–Vigorous PA (mins/day) ^A^	422	707	−0.50	(−1.37, 0.38)	−0.48	(−1.35, 0.40)	−0.29	(−0.93, 0.35)	−0.34	(−0.99, 0.30)
Screen Time (mins/day) ^A^	422	708	0.01	(−0.20, 0.23)	0.02	(−0.20, 0.23)	0.04	(−0.13, 0.21)	0.05	(−0.12, 0.21)
Alcohol intake										
Ever drunk alcohol	424	713	−0.25	(−0.94, 0.44)	−0.20	(−0.90, 0.49)	−0.21	(−0.65, 0.22)	−0.24	(−0.68, 0.20)
Binge drinking	423	709	−0.27	(−0.90, 0.35)	−0.29	(−0.92, 0.33)	−0.10	(−0.56, 0.36)	−0.11	(−0.57, 0.36)
Total weekly drinks	424	713	−0.02	(−0.07, 0.03)	−0.02	(−0.07, 0.04)	0.00	(−0.03, 0.03)	0.00	(−0.04, 0.03)
Sleep behaviors, mean (SD)										
Enough sleep	423	707	0.02	(−0.1, 0.15)	0.02	(−0.1, 0.15)	0.06	(−0.04, 0.16)	0.05	(−0.04, 0.15)
Woke up too early	423	707	0.00	(−0.12, 0.12)	−0.01	(−0.13, 0.11)	0.01	(−0.08, 0.11)	0.01	(−0.08, 0.11)
Tired during day	423	707	−0.01	(−0.13, 0.10)	−0.01	(−0.13, 0.10)	−0.04	(−0.13, 0.05)	−0.04	(−0.12, 0.05)
Hours of sleep on weekdays	424	713	0.01	(−0.15, 0.17)	0.01	(−0.15, 0.17)	0.03	(−0.10, 0.15)	0.03	(−0.10, 0.15)
Hours of sleep on weekends	424	713	0.04	(−0.09, 0.17)	0.04	(−0.09, 0.17)	0.00	(−0.10, 0.10)	0.00	(−0.11, 0.10)
Mental Health, mean (SD)										
Stress	423	712	0.04	(−0.07, 0.16)	0.04	(−0.07, 0.16)	−0.05	(−0.13, 0.03)	−0.05	(−0.13, 0.03)
Depression	423	712	−0.06	(−0.44, 0.32)	−0.06	(−0.44, 0.32)	−0.21	(−0.47, 0.05)	−0.20	(−0.46, 0.07)
Food Security status, n (%)										
Food insecure	423	712	−0.12	(−0.67, 0.42)	−0.14	(−0.69, 0.40)	0.01	(−0.40, 0.42)	0.00	(−0.41, 0.41)

^A^ The variable values were divided by 100 to scale up the beta co-efficient. ^B^ Waist to height ratio was multiplied by 100 to scale up the beta co-efficient. Model A does not control for student demographics. Model B controlled for students’ sex, race/ethnicity, Pell Grant status, and year in college. F/V: Fruits and vegetables; SSB: Sugar Sweetened Beverages; PA: Physical Activity; npid: number of participants; nresp: number of responses. Bolded text indicates statistical significance.

## Supplementary Materials

The following are available online at https://www.mdpi.com/2072-6643/11/9/1996/s1, S1: Summary of SPARC survey items and time points assessed for the current study.
