# Supplementary material for: Evaluating Potential Behavioral Mediators for Increasing Similarity in Friends’ Body Size among College Students
Source: Nutrients. 2019 Aug 23;11(9):1996. doi: 10.3390/nu11091996 (PMC6770838; doi:10.3390/nu11091996)
Supplement: Supplementary file 1 [file nutrients-11-01996-s001.pdf]

S1. Social impact of Physical Activity and nutRition in College (SPARC) study survey questions for college students living in residence halls (2015-2016)

Construct	Q No.	Item	Responses	Variable name (coding)	Source		Time points assessed [A]			
							T1	T2	T3	T4
Demographics	D1	To begin the survey, please enter the participant ID given to you by the researchers below:	Text entry	pid (num)	Created		X	C	C	C
	D2	Please provide us with your first and last name along with your email address. **Your Amazon gift card(s) will be sent over email so <u>please make sure to give us the best email to reach you at</u>.**	First name: _______________	d_first (string)	Created		X	C	C	C
			Last name: _______________	d_last (string)			X			
			Email address:_____________	d_email (string)			X			
	D3	What is your gender?	1. Male<br>2. Female<br>3. Transgender	d_genFMT<br>d_genFMT_num (1-3)	Created		X	X		
		What is your Sex?	1. Male<br>0. Female<br>[For phases 1-2, derived from d_genFMT, T coded to the sex as reported elsewhere]	d_gen<br>d_gen_num (0/1)			X	X	X	X
	D5	How do you usually describe yourself? (check all that apply)	1. White<br>2. Black or African American<br>3. Hispanic or Latino/a<br>4. Asian or Pacific Islander<br>5. American Indian or Alaska Native<br>6. Some other race (please specify): __________	d_white (0/1)<br>d_black (0/1)<br>d_hisp (0/1)<br>d_asian (0/1)<br>d_amna (0/1)<br>d_raceoth (0/1)<br>d_raceothtxt (string)	[1] Neumark-Sztainer, 2002		X			
		[redacted]	Race/ethnicity (to use as a covariate in a model)<br>1. White<br>2. Black<br>3. Hispanic<br>4. Other	d_raceeth4<br>d_raceeth4_num (1-4)			X	C	C	C
	CL2	What is your year in college?	1. 1st year undergraduate<br>2. 2nd year undergraduate<br>3. 3rd year undergraduate<br>4. 4th year undergraduate<br>5. 5th year or more undergraduate	cl_year (1-5)	Created		X			
	CL7	Are you a Pell Grant recipient?	1. Yes<br>2. No<br>3. I do not know ("No" and "I don't know" combined)	cl_pell2 (0/1)	Created		X	C	X	C
	CL10	Please answer the following questions about your living situation.								

		CL 10.1	Have **you** moved since the last time you took this survey?	1. Yes<br>0. No	cl_moved (0/1)	Created			X	X	X
		CL 10.2	Has your **roommate** moved since the last time you took this survey?		cl_roommatemoved (0/1)				X	X	X
	CL11	Which dorm do you live in? (Only asked at times 2-4 if indicated that they moved)			cl_dormqualtrics<br>cl_dormqualtrics_num (1-20)<br>cl_dormqualtricsothtxt (string)	[4] ASU		X	X	X	X
		[redacted]			cl_dorm17<br>cl_dorm17_num (1-17)<br>[derived from cl_dormqualtrics, housing doc, and intake form]			X	C	C	C
	CL12	What is your floor and room/suite number in (fill in dorm from above)? (Only asked at times 2-4 if indicated that they moved)									
		CL12.1	Floor #	Type in: Floor number	cl_dormfloorqualtrics (num)	Created		X	X	X	X
				[redacted]	cl_floor (num)<br>[derived from cl_floorqualtrics, housing doc, and intake form]			X	C	C	C
		CL12.2	Room/suite #	Type in: Room/suite number	cl_dormroomqualtrics (num)			X	X	X	X
				[redacted]	cl_room (num)<br>[derived from cl_roomqualtrics, housing doc, and intake form]			X	C	C	C
		[redacted]		Current dorm<br>(list of dorms same as for "DORM" variable	cl_currentdorm17<br>cl_currentdorm17_num (1-16)				X	X	X
				Current floor	cl_currentfloor (num)				X	X	X
				Current room	cl_currentroom (num)				X	X	X
	CL13	Do you have a roommate? (Only asked at times 2-4 if indicated that they or their roommate moved)		1. Yes, I have a roommate (please list first and last name): ___________________<br>0. No, I do not have a roommate	cl_roommate (0/1)<br>cl_roommatenametxt (string)	Created		X	X	X	X
								X	X	X	X
				Converting name text to:<br>5 digit roommate identifier	cl_roommatepid (num)			X	X	X	X
								X	X	X	X
	FR3	Please rank your top 5 **male** and top 5 **female** friends **at ASU** (the first being your best friend, the									

		second being your next closest friend, and so on).									
		FR3.1	Male	1. First name, Last name	fr_male1_first (string)<br>fr_male2_first (string)<br>fr_male3_first (string)<br>fr_male4_first (string)<br>fr_male5_first (string)<br>fr_male1_last (string)<br>fr_male2_last (string)<br>fr_male3_last (string)<br>fr_male4_last (string)<br>fr_male5_last (string)	[11] Adapted from: Holahan, 1978<br><br>[12]Adapted from: Paxton, 1999<br><br>[13] Adapted from: Wang, 2006<br><br>[14] Adapted from: Add Health, 2009		X	X	X	X
		FR3.2	Female		fr_female1_first (string)<br>fr_female2_first (string)<br>fr_female3_first (string)<br>fr_female4_first (string)<br>fr_female5_first (string)<br>fr_female1_last (string)<br>fr_female2_last (string)<br>fr_female3_last (string)<br>fr_female4_last (string)<br>fr_female5_last (string)						
		[redacted]		Replace alter names with PID/identifier code	fr_male1pid<br>fr_male2pid<br>fr_male3pid<br>fr_male4pid<br>fr_male5pid<br>fr_female1pid<br>fr_female2pid<br>fr_female3pid<br>fr_female4pid<br>fr_female5pid			X	X	X	X
	FR4	For each friend, please provide the dorm they live in. (for each 10 friends)									
		FR4.1	Dorm		fr_maledorm1<br>fr_maledorm2<br>fr_maledorm3<br>fr_maledorm4<br>fr_maledorm5<br>fr_femaledorm1<br>fr_femaledorm2<br>fr_femaledorm3<br>fr_femaledorm4<br>fr_femaledorm5	[4] ASU		X	X	X	X

					fr_maledorm1_num (1-22)<br>fr_maledorm2_num (1-22)<br>fr_maledorm3_num (1-22)<br>fr_maledorm4_num (1-22)<br>fr_maledorm5_num (1-22)<br>fr_femaledorm1_num (1-22)<br>fr_femaledorm2_num (1-22)<br>fr_femaledorm3_num (1-22)<br>fr_femaledorm4_num (1-22)<br>fr_femaledorm5_num (1-22)						
		FR 4.2	Dorm floor	1. Dropdown: 1st-15th floor, does NOT live in the dorms, I do not know	fr_malefloor1 (1-17)<br>fr_malefloor2 (1-17)<br>fr_malefloor3 (1-17)<br>fr_malefloor4 (1-17)<br>fr_malefloor5 (1-17)<br>fr_femalefloor1 (1-17)<br>fr_femalefloor2 (1-17)<br>fr_femalefloor3 (1-17)<br>fr_femalefloor4 (1-17)<br>fr_femalefloor5 (1-17)	Created		X	X	X	X
		[redacted]									
		FR5.3	Roommate	Dropdown<br>1. Yes<br>0. No	fr_maleroomate1 (0/1)<br>fr_maleroomate2 (0/1)<br>fr_maleroomate3 (0/1)<br>fr_maleroomate4 (0/1)<br>fr_maleroomate5 (0/1)<br>fr_femaleroomate1 (0/1)<br>fr_femaleroomate2 (0/1)<br>fr_femaleroomate3 (0/1)<br>fr_femaleroomate4 (0/1)<br>fr_femaleroomate5 (0/1)	Created		X	X	X	X
Stress	SD1	How often in the past <u>1 month</u> have you felt:									
		SD1.1	Unable to control the important things in your life?	1. Never<br>2. Rarely<br>3. Sometimes	sd_control (1-4)	[20] Adapted from: Cohen, 1983		X	X	X	X

		SD1.2	Confident about your ability to handle your personal problems?	4. Often	sd_confident (1-4) [backcoded]			X	X	X	X
		SD1.3	Things were going your way?		sd_yourway (1-4) [reverse coded]			X	X	X	X
		SD1.4	Difficulties were piling up so high that you could not overcome them?		sd_difficult (1-4)			X	X	X	X
				Stress scale (sum SD1.1 – SD1.4)	sd_stressscale (4-16)			X	X	X	X
				Stress scale Cronbach's alpha				.68	.73	.75	.72
Depression		How often in the past <u>1 month</u> have you felt:									
		SD1.5	Things were hopeless?	1. Never<br>2. Rarely<br>3. Sometimes<br>4. Often	sd_hopeless (1-5)	[21] Adapted from: ACHA, 2013		X	X	X	X
		SD1.6	Overwhelmed by all you had to do?		sd_overwhelmed (1-5)			X	X	X	X
		SD1.7	Very lonely?		sd_lonely (1-5)			X	X	X	X
		SD1.8	Very sad?		sd_sad (1-5)			X	X	X	X
		SD1.9	So depressed that it was difficult to function?		sd_depressed (1-5)			X	X	X	X
		SD1.10	Overwhelming anxiety?		sd_anxiety (1-5)			X	X	X	X
				Depression scale Cronbach's alpha				.89	.89	.90	.90
				Depression scale (dichotomized)<br>1. High levels depressive symptoms<br>0. Low levels depressive symptoms	sd_depressionscale2 (0/1)			X	X	X	X
Food Security	FS1	Please indicate how often each statement was true for you in the last <u>1 month</u>:									
		FS1.1	The food that I bought just didn't last, and I didn't have money to get more	1. Often true<br>2. Sometimes true<br>3. Never true	fs_notlast<br>fs_notlast_num (1-3)	[23] Adapted from: ERS		X	X	X	X
		FS1.2	I couldn't afford to eat balanced meals		fs_afford<br>fs_afford_num (1-3)			X	X	X	X
	FS2	In the past <u>1 month</u>, did you ever cut the size of your meals or skip meals because there wasn't enough money for food?		1. No<br>2. Yes, only 1 or 2 weeks<br>3. Yes, some weeks but not every week<br>4. Yes, almost every week	fs_cut<br>fs_cut_num (1-4)	[23] Adapted from: ERS		X	X	X	X
	FS3	Please indicate if each statement was true for you in the last <u>1 month</u>:									

		FS3.1	Did you ever eat less than you felt you should because there wasn't enough money for food?	1. Yes<br>0. No		fs_less (0/1)	[23]<br>Adapted from: ERS		X	X	X	X
		FS3.2	Were you ever hungry but didn't eat because there wasn't enough money for food?			fs_hunger (0/1)			X	X	X	X
				Food insecure (Selected "yes" to at least 2 of the above questions)<br>1. Yes<br>0. No		fs_fi<br>fs_fi_num (0/1)			X	X	X	X
Eating Behaviors	EB1	In the past <u>7 days</u>, how often did you eat the following:										
		EB1.1	Breakfast?	0. Never<br>1. 1 day<br>2. 2 days<br>3. 3 days<br>4. 4 days<br>5. 5 days<br>6. 6 days<br>7. 7 days		eb_breakfast (0-7)*	*[24] Adapted from Larson, 2012<br><br>**[25] Adapted from Rodriguez<br><br>***[26] Adapted from Laska, 2014		X	X	X	X
		EB1.2	Evening meal?			eb_evening (0-7)***			X	X	X	X
		EB1.3	Fast foods (e.g., McDonald's, Raising Canes, Taco Bell, Dominos, Panda Express, etc.)?			eb_fastfood (0-7)*			X	X	X	X
		EB1.4	Sit-down restaurant food (e.g., Olive Garden, Oreganos, etc.)?			eb_restaurant (0-7)**			X	X	X	X
		EB1.5	Dining hall food?			eb_dininghall (0-7)**			X	X	X	X
		EB1.6	Home-cooked foods (e.g., meals made from scratch)?			eb_homecook (0-7)**			X	X	X	X
Eating Behaviors: Diet Screener	DS1	During the <u>past month</u>, how often did you eat **hot or cold cereals**? (if Never selected skip next 2 questions)		Never<br>1 time last month<br>2-3 times last month<br>1 time per week<br>2 times per week<br>3-4 times per week<br>5-6 times per week<br>1 time per day<br>2 or more times per day	0 times/d<br>0.033 times/d<br>0.083 times/d<br>0.143 times/d<br>0.286 times/d<br>0.5 times/d<br>0.786 times/d<br>1 times/d<br>2 times/day	ds_cereal	[28] NHANES 2009-2010		X	X	X	X
	DS2	During the <u>past month</u>, what kind of cereal did you usually eat? – *Type cereal*		Text entry		ds_cerealtxt (string)	[28] NHANES 2009-2010		X	X	X	X
	DS3	If there was another kind of cereal that you usually ate during		Text entry		ds_cerealtxt2 (string)	[28] NHANES 2009-2010		X	X	X	X

		the past month, what kind was it?<br>*–Type cereal, if none leave blank*									
		[redacted]	NIH 8 digit code for cereal 1 & 2<br>(indicates the tertile classification for added sugar, whole grain density, fiber density, and calcium density)		ds_c1code<br>ds_c2code			X	X	X	X
			Added sugar classification for cereal 1 & 2<br>(tsp added sugar/100 grams)<br>1. Lowest tertile: ≤0.71<br>2. Second tertile: 0.72-5.49<br>3. Highest tertile: >5.49		ds_c1sugnt (1-3)<br>ds_c2sugnt (1-3)			X	X	X	X
			Whole grain density classification for cereal 1 &2 (oz-equivalents of whole grains/100 grams)<br>1. Lowest tertile: ≤0.21<br>2. Second tertile: 0.22-1.40<br>3. Highest tertile: >1.40		ds_c1whgnt (1-3)<br>ds_c2whgnt (1-3)			X	X	X	X
			Calcium density classification for cereal 1 &2<br>(mg of calcium/100 grams)<br>1. Lowest tertile: ≤21<br>2. Second tertile: 22-100<br>3. Highest tertile: >100		ds_c1calcnt (1-3)<br>ds_c2calcnt (1-3)			X	X	X	X
		[redacted]	Fiber density classification for cereal 1 &2<br>(grams of fiber/100 grams)<br>1. Lowest tertile: ≤2.1<br>2. Second tertile: 2.2-7.3<br>3. Highest tertile: >7.3		ds_c1fibnt (1-3)<br>ds_c2fibnt (1-3)			X	X	X	X
	DS4	During the past month, how often did you have any **milk** (either to drink or on cereal)? Include regular milks, chocolate, or other flavored milks, lactose-free milk, buttermilk and soy. Please do **not** include small amounts of milk in coffee or tea. (If Never is selected skip next question)	Never<br>1 time last month<br>2-3 times last month<br>1 time per week<br>2 times per week<br>3-4 times per week<br>5-6 times per week<br>1 time per day<br>2-3 times per day<br>4-5 times per day<br>6 or more times per day	0 times/d<br>0.033 times/d<br>0.083 times/d<br>0.143 times/d<br>0.286 times/d<br>0.5 times/d<br>0.786 times/d<br>1 time/d<br>2.5 times/d<br>4.5 times/d<br>6 times/d	ds_milk	[28] NHANES 2009-2010		X	X	X	X
	DS5	During the past month, what kind of milk did you usually drink?	1. Whole or regular milk<br>2. 2% or reduced-fat milk<br>3. 1% or low-fat milk<br>4. Fat-free, skim or nonfat milk<br>5. Soy milk<br>6. Other kind of milk- *Type milk* ____________<br>7. Almond (added in after backcoding)		ds_milktype<br>ds_milktype_num (1-7)<br>ds_milktypeothtxt (string)	[28] NHANES 2009-2010		X	X	X	X
	DS6	During the past month, how often did you drink:									

		DS6.1	**Regular** soda or pop that contains sugar? *Do **not** include diet soda.*	Never<br>1 time last month<br>2-3 times last month<br>1 time per week<br>2 times per week<br>3-4 times per week<br>5-6 times per week<br>1 time per day<br>2-3 times per day<br>4-5 times per day<br>6 or more times per day	0 times/d<br>0.033 times/d<br>0.083 times/d<br>0.143 times/d<br>0.286 times/d<br>0.5 times/d<br>0.786 times/d<br>1 time/d<br>2.5 times/d<br>4.5 times/d<br>6 times/d	ds_soda	[28] NHANES 2009-2010<br><br>[29] CCS 2010		X	X	X	X
		DS6.2	**100% pure fruit juices** such as orange, mango, apple, grape and pineapple juices? *Do **not** include fruit-flavored drinks with added sugar or fruit juice you made at home and added sugar to.*			ds_juice			X	X	X	X
		DS6.3	Coffee or tea that had **sugar** or **honey** added to it? Include coffee and tea you sweetened yourself and presweetened tea and coffee drinks such as Arizona Tea and Frappuccino. *Do **not** include artificially sweetened coffee or diet tea.*			ds_coffee			X	X	X	X
		DS6.4	**Sweetened fruit drinks** such as Kool-Aid, Lemonade, or cranberry drink? Include fruit drinks you made at home and added sugar to. *Do **not** include diet drinks or artificially sweetened drinks.*			ds_fruitdrink			X	X	X	X
		DS6.5	**Sports drinks** such as Gatorade, Powerade, Vitamin Water, etc.? *Do **not** include diet drinks or artificially sweetened drinks.*			ds_sportdrink			X	X	X	X
		DS6.6	**Energy drinks** such as Red Bull, Monster, Rockstar, etc.? *Do **not** include diet*			ds_energydrink			X	X	X	X

			drinks or artificially sweetened drinks.									
	DS7	During the <u>past month</u>, how often did you eat:										
		DS7.1	**Fruit**? Include fresh, frozen or canned fruit. *Do **not** include juices.*	Never<br>1 time last month<br>2-3 times last month<br>1 time per week<br>2 times per week<br>3-4 times per week<br>5-6 times per week<br>1 time per day<br>2 or more times per day	0 times/d<br>0.033 times/d<br>0.083 times/d<br>0.143 times/d<br>0.286 times/d<br>0.5 times/d<br>0.786 times/d<br>1 time/d<br>2 times/d	ds_fruit	[28] NHANES 2009-2010		X	X	X	X
		DS7.2	Green leafy or lettuce **salad**, with or without other vegetables?			ds_salad			X	X	X	X
		DS7.3	Any kind of **fried potatoes**, include French fries, home fries, or hash brown potatoes?			ds_friedpotato			X	X	X	X
		DS7.4	Any **other kind of potatoes**, such as baked, boiled, mashed, sweet potatoes, or potato salad?			ds_othpotato			X	X	X	X
		DS7.5	Refried beans, baked beans, beans in soup, pork and beans or any other type of cooked dried beans? *Do **not** include green beans.*			ds_beans			X	X	X	X
		DS7.6	**Brown rice** or other cooked whole grains, such as bulgur, cracked wheat, or millet? *Do **not** include white rice.*			ds_rice			X	X	X	X
		DS7.7	**Other vegetables** (not including previously mentioned green salads, potatoes, or cooked dried beans)?			ds_othveg			X	X	X	X
		DS7.8	Mexican-type **salsa** made with tomato?			ds_salsa			X	X	X	X
	DS8	During the <u>past month</u> how often did you eat:										

		DS8.1	**Pizza**? Including frozen pizza, fast food pizza, and homemade pizza.	Never<br>1 time last month<br>2-3 times last month<br>1 time per week<br>2 times per week<br>3-4 times per week<br>5-6 times per week<br>1 time per day<br>2 or more times per day	0 times/d<br>0.033 times/d<br>0.083 times/d<br>0.143 times/d<br>0.286 times/d<br>0.5 times/d<br>0.786 times/d<br>1 time/d<br>2 times/d	ds_pizza	[28] NHANES 2009-2010		X	X	X	X
		DS8.2	**Tomato sauces** such as with spaghetti noodles or mixed into foods such as lasagna? *Do **not** include tomato sauce on pizza.*			ds_sauce			X	X	X	X
		DS8.3	Any kind of **cheese**? Include cheese as a snack, cheese on burgers, sandwiches, and cheese in foods such as lasagna, quesadillas, or casseroles. *Do **not** include cheese on pizza.*			ds_cheese			X	X	X	X
		DS8.4	**Red meat**, such as beef, pork, ham, or sausage? Include red meat you had in sandwiches, lasagna, stew and other mixtures. Red meats may also include veal, lamb, and any lunch meats made with these meats. *Do **not** include chicken, turkey or seafood.*			ds_redmeat			X	X	X	X
		DS8.5	**Processed meat**, such as bacon, lunch meats, or hot dogs? Include processed meats you had in sandwiches, soups, pizza, casseroles, and other mixtures. Processed meats are those preserved by smoking, curing, or salting, or by the			ds_processed			X	X	X	X

			addition of preservatives. Examples are: ham, bacon, pastrami, salami, sausages, bratwursts, frankfurters, hot dogs, and spam.									
	DS9	During the <u>past month</u>, how often did you eat:										
		DS9.1	**Whole grain bread** including toast, rolls and in sandwiches? Whole grain breads include whole wheat, rye, oatmeal, pumpernickel. *Do **not** include white bread.*	Never<br>1 time last month<br>2-3 times last month<br>1 time per week<br>2 times per week<br>3-4 times per week<br>5-6 times per week<br>1 time per day<br>2 or more times per day	0 times/d<br>0.033 times/d<br>0.083 times/d<br>0.143 times/d<br>0.286 times/d<br>0.5 times/d<br>0.786 times/d<br>1 time/d<br>2 times/d	ds_grain	[28] NHANES 2009-2010		X	X	X	X
		DS9.2	**Chocolate** or any other types of candy? *Do **not** include sugar-free candy.*			ds_chocolate			X	X	X	X
		DS9.3	**Doughnuts**, sweet rolls, Danish, muffins, pan dulce, or pop-tarts? *Do **not** include sugar-free items.*			ds_doughnut			X	X	X	X
		DS9.4	**Cookies**, cakes, pie or brownies? *Do **not** include sugar-free kinds.*			ds_cookie			X	X	X	X
		DS9.5	**Ice cream** or other frozen desserts? *Do **not** include sugar-free kinds.*			ds_icecream			X	X	X	X
		DS9.6	**Popcorn**?			ds_popcorn			X	X	X	X
				Predicted intake of fiber (gm) per day		DSQfib	Created		X	X	X	X
				Predicted intake of calcium (mg) per day		DSQcalc	Created		X	X	X	X
				Predicted intake of whole grains (ounce equivalents) per day		DSQwhgr	Created		X	X	X	X
				Predicted intake of total added sugars (tsp equivalents) per day		DSQsug	Created		X	X	X	X
				Predicted intake of dairy (cup equivalents) per day		DSQdairy	Created		X	X	X	X
				Predicted intake of fruits and vegetables including legumes and French fries (cup equivalents) per day		DSQfvl	Created		X	X	X	X

	[redacted]			Predicted intake of vegetables including legumes and French fries (cup equivalents) per day		DSQvlall	Created		X	X	X	X
				Predicted intake of fruits and vegetables including legumes and excluding French fries (cup equivalents) per day		DSQfvlnf	Created		X	X	X	X
				Predicted intake of vegetables including legumes and excluding French fries (cup equivalents) per day		DSQvlnf	Created		X	X	X	X
				Predicted intake of fruits (cup equivalents) per day		DSQfrt	Created		X	X	X	X
				Predicted intake of added sugars from sugar-sweetened beverages (tsp equivalents) per day		DSQssb	Created		X	X	X	X
Alcohol			Have you <u>ever</u> drank alcohol?	1. Yes<br>0. No		a_drinkalch (0/1)	Created		X	X	X	X
			For each day of the week in the calendar below, indicate the **number of alcoholic drinks <u>typically consumed</u>** on that day. (Only if yes to alcohol is selected)	Dropdown from 1-15 drinks:<br>Monday<br>Tuesday<br>Wednesday<br>Thursday<br>Friday<br>Saturday<br>Sunday		a_drinkmon (num)<br>a_drinktue (num)<br>a_drinkwed (num)<br>a_drinkthur (num)<br>a_drinkfri (num)<br>a_drinksat (num)<br>a_drinksun (num)	[34]<br>Kruse, 2005		X	X	X	X
		[redacted]		Converting reported drinks into number of drinks/week		a_totweeklydrinks (num)			X	X	X	X
	A3		During the last <u>two weeks</u>, how many times have you had **<u>four</u> alcoholic drinks in a row**? (Only if female selected) (Only if yes to alcohol is selected)	Never<br>1 day<br>2 days<br>3 days<br>4 or more days	0<br>1<br>2<br>3<br>4	a_fourrow (0-4)	[35] Adapted from: Wechsler, 1994		X	X	X	X
	A4		During the last <u>two weeks</u>, how many times have you had **<u>five</u> alcoholic drinks in a row**? (Only if not female selected) (Only if yes to alcohol is selected)			a_fiverow (1-5)			X	X	X	X
		[redacted]		Binge drinker? (4 or 5 alcoholic drinks in a row for females and males, respectively)<br>1. Yes<br>0. No		a_bingedrink (0/1)			X	X	X	X
Sleep	SL1		In the <u>past 7 days</u>, how often did the following occur?									
		SL1.1	You got enough sleep so that you felt rested when you woke up in the morning	0. 0 days<br>1. 1 day<br>2. 2 days<br>3. 3 days<br>4. 4 days<br>5. 5 days<br>6. 6 days		sl_enough (0-7)	[21] Adapted from: ACHA, 2013		X	X	X	X
		SL1.2	You woke up too early in the morning			sl_wokeup (1-8)			X	X	X	X

			and couldn’t get back to sleep	7. 7 days								
		SL1.3	You felt tired, dragged out, or sleepy during the day			sl_tired (1-8)			X	X	X	X
	SL2	These next questions ask about your sleep patterns.										
		SL2.2	On an **<u>average weekday,</u>** how many **hours** of sleep do you usually get?	Dropdown (1-16 hours) (with half hour increments)		sl_wkdayhrs	Created		X	X	X	X
		SL2.4	On an **<u>average weekend day</u>** how many **hours** of sleep do you usually get?	Dropdown (1-16 hours) (with half hour increments)		sl_wkendhrs	Created		X	X	X	X
Physical Activity	PA1	In a **<u>usual week</u>**, how many hours do **<u>you</u>** spend doing the following activities:										
		PA1.1	**Strenuous exercise (heart beats rapidly)**? Examples: biking fast, aerobic dancing, running, jogging, swimming laps, rollerblading, skating, lacrosse, tennis, cross-country skiing, soccer, basketball, football, zumba	None<br>Less than 1/2 hour a week<br>1/2-2 hours a week<br>2 1/2-4 hours a week<br>4 1/2-6 hours a week<br>More than 6 hours a week	0 min<br>30 min<br>75 min<br>195 min<br>315 min<br>360 min	pa_vig (1-6)	[39] Godin, 1985		X	X	X	X
		PA1.2	**Moderate exercise (not exhausting)?** Examples: walking quickly, baseball, gymnastics, easy bicycling, volleyball, skiing, dancing, skateboarding, snowboarding			pa_mod (1-6)			X	X	X	X
		PA1.3	**Mild exercise (little effort)?** Examples: walking slowly (to school, to friend’s house, etc.), bowling, golf, fishing, snowmobiling, yoga			pa_light (1-6)			X	X	X	X

			Moderate-to-vigorous physical activity	pa_mvpa (num)			X	X	X	X
	PA3	**<u>Yesterday</u>**, how much time did you spend in front of a screen (excluding time in class and being physically active)? This includes computers, tablets, smartphones, TV, video games, movies, etc.	1. None<br>2. Less than ½ hour<br>3. ½- 2 hours<br>4. 2 ½- 4 hours<br>5. 4 ½-6 hours<br>6. More than 6 hours	pa_screen (1-6)	[41] Adapted from: Fountaine, 2011		X	X	X	X

[A] X= asked at that time point or derived from a question asked at that time point; C= carried over from another time point into data set

Citations

1. Neumark-Sztainer D, Croll J, Story M, Hannan PJ, French SA, Perry C. Ethnic/racial differences in weight-related concerns and behaviors among adolescent girls and boys: findings from Project EAT. *Journal of psychosomatic research.* 2002;53(5):963-974.

4. Arizona State University. Tempe Campus. https://housing.asu.edu/content/tempe-campus. Accessed 2014, 16 July.

11. Holahan CJ, Wilcox BL. Residential satisfaction and friendship formation in high-and low-rise student housing: An interactional analysis. *Journal of Educational psychology.* 1978;70(2):237.

12. Paxton SJ, Schutz HK, Wertheim EH, Muir SL. Friendship clique and peer influences on body image concerns, dietary restraint, extreme weight-loss behaviors, and binge eating in adolescent girls. *Journal of abnormal psychology.* 1999;108(2):255.

13. Wang SS, Houshyar S, Prinstein MJ. Adolescent girls' and boys' weight-related health behaviors and cognitions: associations with reputation-and preference-based peer status. *Health Psychology.* 2006;25(5):658.

14. Harris KM FF, Tabor J, Bearman PS, Jones J, Udry JR. The National Longitudinal Study of Adolescent Health: Research Design. Accessed May 2007.

20. Cohen S, Kamarck T, Mermelstein R. A global measure of perceived stress. *Journal of health and social behavior.* 1983:385-396.

21. American College Health Association-National College Health Assessment II. Hanover, MD: American College Health Association; 2013.

23. U.S. Household Food Security Survey Module: Six-Item Short Form Economic Research Service, USDA. 2012; http://www.ers.usda.gov/datafiles/Food_Security_in_the_United_States/Food_Security_Survey_Modules/short2012.pdf. Accessed 13 April 2015.

24. Larson N, Laska MN, Story M, Neumark-Sztainer D. Predictors of fruit and vegetable intake in young adulthood. *Journal of the Academy of Nutrition and Dietetics.* 2012;112(8):1216-1222.

25. Rodriguez J. Resident Freshmen Diet and Exercise Survey. http://www.unf.edu/~jrodrigu/survey.htm Accessed 17 July, 2014.

26. Laska MN, Hearst MO, Lust K, Lytle LA, Story M. How we eat what we eat: identifying meal routines and practices most strongly associated with healthy and unhealthy dietary factors among young adults. *Public health nutrition.* 2014:1-11.

28. National Cancer Institute. Dietary Screener Questionnaire in the NHANES 2009-10. 2009; http://appliedresearch.cancer.gov/nhanes/dietscreen/dsq_english.pdf. Accessed 22 June, 2015.

29. National Cancer Institute. Dietary Screener Questionnaire in the NHIS Cancer Control Supplement (CCS) 2010. 2010; http://healthcaredelivery.cancer.gov/nhis/2010-screener/. Accessed 22 June, 2015.

34. Kruse M, Corbin W, Fromme K. Improving accuracy of QF measures of alcohol use: Disaggregating quantity and frequency. Paper presented at: Poster presented at the 28th annual meeting of the Research Society on Alcoholism, Santa Barbara, CA2005.

35. Wechsler H, Davenport A, Dowdall G, Moeykens B, Castillo S. Health and behavioral consequences of binge drinking in college: A national survey of students at 140 campuses. *Jama.* 1994;272(21):1672-1677.

39. Godin G, Shephard RJ. A simple method to assess exercise behavior in the community. *Canadian journal of applied sport sciences Journal canadien des sciences appliquees au sport.* 1985;10(3):141-146.

41. Fountaine CJ, Liguori GA, Mozumdar A, Schuna Jr JM. Physical activity and screen time sedentary behaviors in college students. *International Journal of Exercise Science.* 2011;4(2):3.
